# A Missed Diagnosis of Bilateral Simultaneous Spontaneous Intracapsular Neck of Femur Fractures in a Rheumatoid Arthritis Patient

**DOI:** 10.1155/2019/4783573

**Published:** 2019-12-27

**Authors:** Aysha Rajeev, Faizan Jabbar, Emadeldeen Zourob, Kailash Devalia

**Affiliations:** Queen Elizabeth Hospital, Gateshead, UK

## Abstract

Bilateral spontaneous simultaneous fractures of the neck of femurs are extremely rare, and only a few cases have been reported in literature. They are usually following high-energy trauma or may be due to an underlying pathological process such as frailty, osteomalacia, rickets, and chronic renal disease. They can also occur following epilepsy and electric shock. We report a 79-year-old gentleman who presented with sudden onset of bilateral hip pain with a background of rheumatoid arthritis and long-term steroid treatment. The bilateral hip fractures were missed initially and later presented with completely displaced fractures of the neck of femurs. He underwent a single-stage bilateral cemented hemiarthroplasty and made a good recovery. Bilateral simultaneous fractures of the hip in patients with rheumatoid arthritis have not been reported in literature so far, and the diagnosis can be easily overlooked. In patients with bilateral hip pain, one should have a high index of suspicion. Further appropriate cross-sectional imaging in the form of CT or MRI should also be considered.

## 1. Introduction

Spontaneous and simultaneous bilateral fractures of the neck of femurs are relatively uncommon. They are usually associated with an underlying pathology or associated medical conditions leading to a reduction in both quantity and quality of the bone. The pathological conditions of the bone associated with bilateral fractures of the hips are osteomalacia [1], renal osteodystrophy [2], rickets [3], and hyperparathyroidism [4]. Evidence also has shown that these fractures can also occur in patients with generalised epilepsy [5] and those who have sustained an electric shock [6]. Therefore, simultaneous bilateral hip fractures can occur in both high- [7] and low-energy trauma [8].

We report a case of bilateral spontaneous and simultaneous fractures of the neck of femur in a patient with severe rheumatoid arthritis on long-term steroid treatment. The patient presented with sudden onset of bilateral hip pain, which was missed on the initial plain radiographs.

## 2. Case Presentation

A 79-year-old gentleman who lived alone and mobilised with a walking frame was seen in his GP surgery complaining of sudden onset bilateral hip pain. He gave a past medical history of rheumatoid arthritis for many years treated with leflunomide and prednisolone. He also suffered from atrial fibrillation and was on warfarin. He described a severe aching type pain within the hips and was unable to weight bear at the time. His Abbreviated Mental Test (AMT) score was 10. The general practitioner ordered a pelvic X-ray and appropriately referred the patient to orthopaedics for further specialist assessment. The X-ray was reviewed by the orthopaedic registrar on call at the time, and an initial diagnosis of an acute exacerbation of rheumatoid arthritis was made. Advice was given to manage the patient with analgesia and anti-inflammatories.

**Figure 1:**
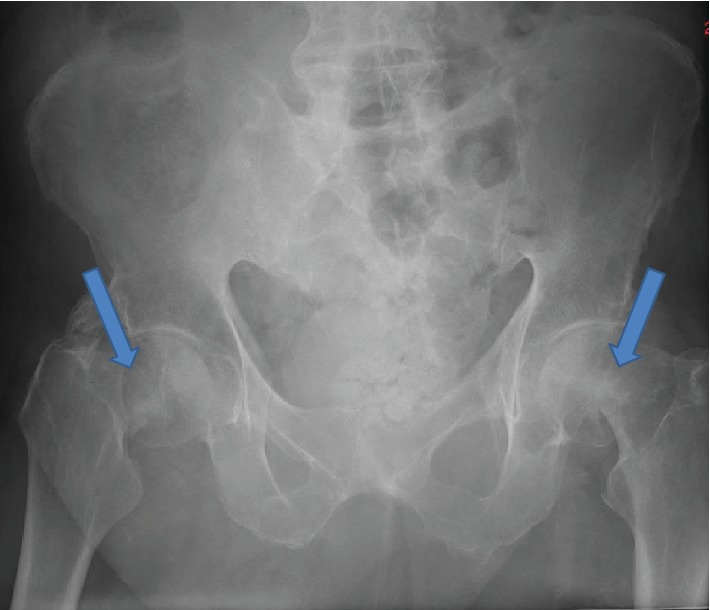
AP view of the pelvis and hips showing bilateral displaced intracapsular fractures.

**Figure 2:**
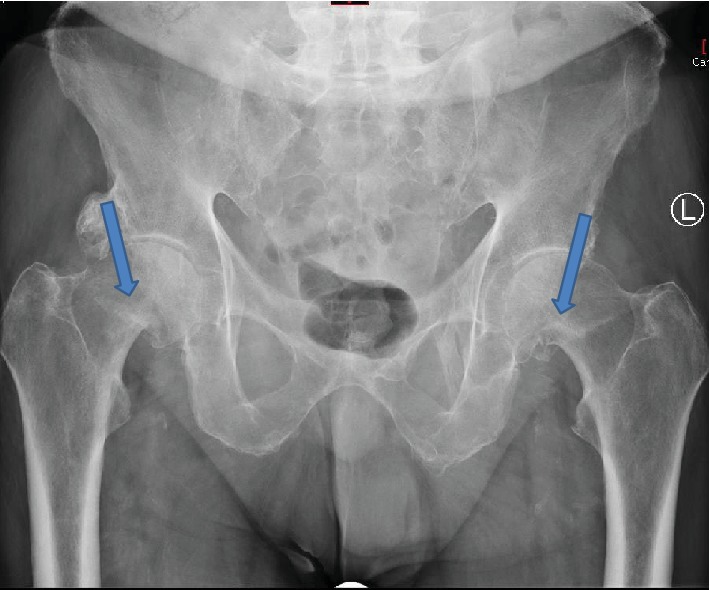
AP view of the pelvis with both hips showing bilateral undisplaced fractures of the neck of femurs.

The patient then attended after ten days when he had a trivial fall due to his “legs giving way.” On clinical examination, he had tenderness in both hips. Both lower limbs were externally rotated and shortened during assessment. Range of motion was extremely limited due to pain. Plain radiographic examination of the pelvis revealed bilateral displaced intracapsular fractures (Figure 1). On reviewing the initial radiographs ordered by the general practitioner, it was apparent that the patient had bilateral undisplaced fractures of the neck of femurs (Figure 2).

**Figure 3:**
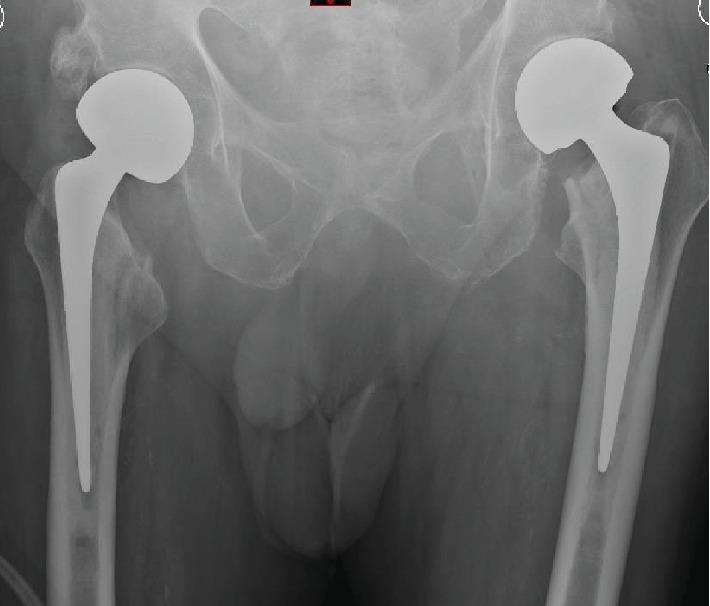
Postoperative radiograph showing bilateral cemented Exeter hemiarthroplasties.

The patient was admitted to the acute trauma ward and optimised by the orthogeriatrician. He then underwent a single-stage bilateral cemented Exeter hemiarthroplasties in lateral position using anterolateral approaches (Figure 3). The patient was transferred to ITU postoperatively and monitored. He was shifted to a rehabilitation ward for intensive physiotherapy. He had an uneventful recovery with mobilisation using a walking frame and was discharged home after 20 days.

## 3. Discussion

Patients who have longstanding rheumatoid arthritis are prone to generalised osteoporosis which increases the risk of fragility fractures in the proximal femurs and vertebra [9]. They are prone to trochanteric fractures as a result of decrease in the cancellous bone and a decrease in cortical bone predisposes to intracapsular fractures of the hip [10]. This process is accelerated if the patients are on long-term steroid treatment along with other antirheumatoid agents [11]. Our patient had chronic rheumatoid arthritis and has been taking prednisolone for many years.

Bilateral fractures of femoral neck of femur can occur as a result of high-velocity injuries [7] or fall from a height [12]. But if there is a structural change to the bone or metabolic bone conditions, these fractures can occur even due to minor or trivial trauma. The conditions predisposing to weakness of the bone are old age and fragility [8], renal osteodystrophy [2], osteomalacia [1], rickets [3], hyperparathyroidism [4], osteoporosis, long-term steroid use, and rheumatoid arthritis. Our patient had two of the aforementioned risk factors.

Another group of patients who are susceptible to these rare fractures are those who suffer from tonic clonic seizures [5] and those undergoing electroconvulsive therapy [6]. The violent contraction of the pelvic trochanteric muscles results in these rare fractures [13]. The antiepileptic medications can alter the bone turnover. This reduces bone mineral density and increases the risk of fracture [14].

The treatment approach for bilateral neck of femur fractures is surgical. The different procedures for the definitive treatment are closed or open reduction with internal fixation, hemi or total hip arthroplasty, and valgus intertrochanteric osteotomy [15]. This can be done either as single- or two-staged procedures [3]. Sood et al. treated these injuries with a single-stage cemented hemiarthroplasty in supine position using anterolateral approaches [16]. McGoldrick et al. advocate single-stage total hip arthroplasty in the lateral position [17]. Specific to our patient, we also performed a single-stage bilateral cemented hemiarthroplasty in the lateral position using an anterolateral approach.

## 4. Conclusion

Simultaneous spontaneous bilateral fractures of the neck of femurs are rare. A diagnosis of fracture neck of femur should be considered in the differential diagnosis for rheumatoid arthritis patients who are on long-term steroid treatment and present with hip pain. In such cases, further imaging investigations such as CT and MRI scans should be considered to exclude an underlying stress or insufficiency fracture of the proximal femora so that early and definitive treatment can be initiated.
